# The lived experiences of intensive care nursing students exposed to a new model of high-fidelity simulation training: a phenomenological study

**DOI:** 10.1186/s12912-021-00667-3

**Published:** 2021-08-30

**Authors:** Angelo Dante, Vittorio Masotta, Alessia Marcotullio, Luca Bertocchi, Valeria Caponnetto, Carmen La Cerra, Cristina Petrucci, Celeste Marie Alfes, Loreto Lancia

**Affiliations:** 1 Department of Health, Life and Environmental Sciences, University of L’Aquila - Rita Levi Montalcini Building, G. Petrini Street, 67100 L’Aquila, Italy; 2 Frances Payne Bolton School of Nursing, Case Western Reserve University - Health Education Office 269B, Cleveland, Ohio USA

**Keywords:** Nursing students, Postgraduate, High-fidelity simulation, Lived experiences, Phenomenological study

## Abstract

**Background:**

In postgraduate intensive care nursing courses, high-fidelity simulation is useful to prepare students to guarantee safe and quality care of critically ill patients. Surprisingly, this issue has not attracted sufficient attention in the literature, and it is not clear whether the linear application of the traditional high-fidelity simulation method based on prebriefing, the simulation session and debriefing, can serve as empirical reference in postgraduate students’ education. The aim of this study was to investigate the lived experiences of postgraduate students receiving multiple exposures to an innovative high-fidelity simulation design based on Kolb’s Experiential Learning Theory.

**Methods:**

A phenomenological study was conducted at an Italian University involving a purposive sample of 15 nursing students attending the postgraduate intensive care course. Audio-recorded face-to-face in-depth interviews were held by a researcher in a dedicated room complemented with non-verbal communication outlined in the field notes. Thematic analysis was used to analyse the transcribed data.

**Results:**

Three themes and ten categories were derived from the data analysis. The themes included pragmatic learning experience, the emotional path, and confidence.

**Conclusions:**

Multiple exposure to high-fidelity simulation was lived as a pragmatic learning experience enhancing the students’ ability to apply theory into practice. This novel approach also contributed to the transition from negative to positive feelings and improved students’ confidence about technical and non-technical skills when caring for a critically ill patient.

## Background

Critical care nurses are required to possess a combination of skills, knowledge, and attitudes useful to improve nursing care of critically ill patients [1]. To obtain advanced technical skills and complex non-technical abilities (e.g., communicate with patients and co-workers, teamwork ability, face end-of-life care and moral issues) required to perform their role, critical care nurses can aspire to attend several academic postgraduate courses worldwide [2, 3]. In these courses, clinical training is traditionally performed at the bedside, on ambulances or in first-responder services and it is generally integrated through different typologies of technology-based educational strategies, that prepares students to face a dynamic healthcare domain characterized by high hazard and invasive interventions [4]. High-fidelity simulation (HFS) is a teaching and learning method based on real clinical scenarios performed in a realistic, safe, and forgiving environment through a technologically advanced human patient simulator (mannequin) able to reproduce life-like clinical conditions, which enables students to acquire technical and non-technical skills [5–7]. The fidelity concept is related to the perception of realism by students and it is positively associated to their engagement into learning activity [8]. Fidelity encompasses the following dimensions: 1) physical, which consists in how the setting closely replicates the clinical environment; 2) conceptual, which consists in ensuring that clinical scenarios make sense and guarantee the consistency between learning goals and students’ needs; 3) psychological, which is related to the contextual elements that can be found in the clinical setting, such as social relationships, distractions, or competing priorities [8, 9]. In accordance with the international standards of best practice in simulation, HFS sessions offered within courses are based on a systematic and cyclical planning which provides prebriefing, simulation-based experience, and debriefing [8]. The linear application of this approach appears to be the most utilized worldwide for its educational effectiveness in improving learning outcomes, especially in undergraduate nursing students [10–12]. However, it is known that the different methods of HFS can affect the magnitude of the learning outcomes and the literature does not clarify the optimal duration of HFS sessions, the ideal number of expositions and participants, the most effective facilitation methods, and whether the traditional HFS method can serve as empirical reference in postgraduate students’ education [6, 12, 13]. Hypothetically, these students have both a higher level of competence and clinical experience than undergraduates and they likely need a different approach in simulation design to obtain higher level learning outcomes than those documented in the literature [6, 14, 15]. Therefore, research efforts to test new HFS modalities that aid the clinical learning process, especially in postgraduate students, are needed. At the University of L’Aquila, located in the centre of Italy, a postgraduate critical care program which incorporates innovative HFS modalities was implemented and evaluated (Table 1).

**Table 1:** Features of Postgraduate Intensive Care Course

**Objectives.** Provide participants with advanced nursing skills for the management of life-threatening conditions.	
**Admission**. An entry exam is required to be admitted.	
**Credits and hours**. 60 credits for a total of 1500 h (1 credit = 25 h).	
**Lectures**. 30 credits. At least 75% in-class presence is mandatory.	
**Clinical training**. 22 credits in Critical Care Area or on rescue vehicles are required. The Simulation Lab allows students to practice on advanced nursing clinical techniques.	
**Exams**. Students need to pass four exams composed by a total of 23 disciplines.	
**Faculty**. Director, lecturers, and clinical tutors as facilitators for students during their clinical training.	
**Final exam**. The discussion of a thesis is required to achieve the Master.	

During the postgraduate critical care program, students are expected to attend several HFS sessions in a laboratory outfitted with a technologically advanced human patient simulator, as well as all medical equipment and devices typically utilized within an Intensive Care Unit to simulate the nursing management of high risk, low incidence life-threatening clinical conditions. In the simulation laboratory is guaranteed a high level of environmental fidelity, while the availability of dedicated expert faculty ensure attention to conceptual and psychological aspects in each simulation session.

Beginning with the 2018–2019 academic year, graduate nursing students enrolled in the postgraduate critical care program received multiple exposures to an innovative HFS design based on the Kolb’s Experiential Learning Theory [16].

**Fig. 1:**
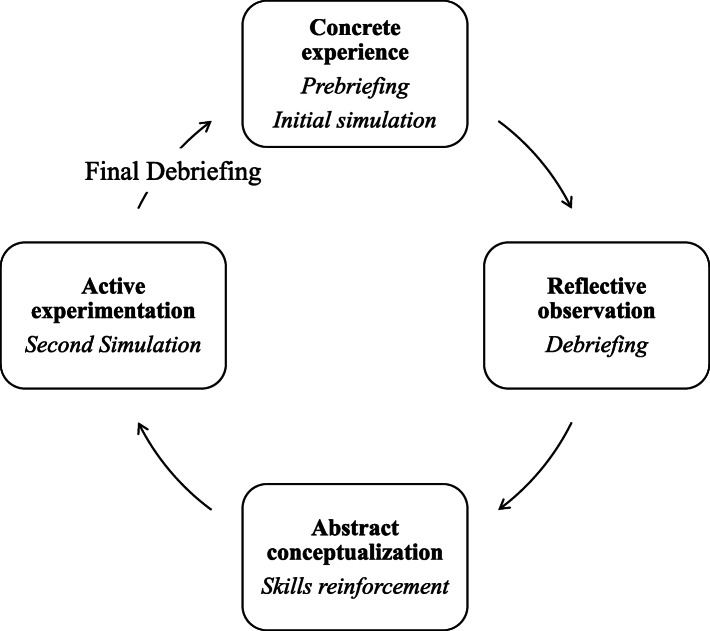
Application of the Kolb’s Theory to the HFS learning experience

Kolb’s Experiential Learning Theory, which describes the cyclical process of learning, was used to structure different phases within the simulation sessions (Fig. 1).

After attending lectures about respiratory diseases and the related clinical management, students took part in their first four-hour HFS experience based on an acute respiratory failure scenario. The ‘concrete experience’ consisted of both prebriefing and simulation in which students were engaged with an acute respiratory failure scenario. The first phase was followed by a video-assisted debriefing in which students reflected on and reviewed their learning experience under the guidance of their teacher (reflective observation). The moment of the supervised skills reinforcement allowed time for the ‘abstract conceptualization’ in which students tried to draw conclusions of the experience by reflecting on their prior knowledge, skills, and attitudes and had the chance to see how what they learned can be applied onto the real world. Finally, students applied what they learned being involved in active experimentation within the second simulation session.

The same four-hour HFS experience was repeated one month apart with worsened and more critical clinical conditions of the simulated patient. Both for the first and the second simulation experience, students were divided into groups of three, with the group composition changing in the second experience based on students’ availability. During the final debriefing for the HFS experiences, faculty gave students the opportunity to enhance their knowledge of safety and quality of care by focusing student’s knowledge on best practices under the guidance of faculty and clinical experts.

The novel learning model based on multiple exposure to the HFS aimed at improving the learning experience of postgraduate students enrolled in a postgraduate critical care course. To know the full experience and perspectives of participants is the first step to fill current literature gaps about the best HFS application modality in postgraduate courses. In addition, the students’ point of view allows faculty to make improvements in the training experience and to ensure consistency with participants’ training needs. For this reason, according to the first level (reaction) of Kirkpatrick’s Learning Evaluation Model [17], this study aimed at investigating the lived experiences of postgraduate students receiving multiple exposures to an innovative HFS design.

## Methods

### Study design

A phenomenological study was conducted with reporting checked against the ‘Consolidated criteria for reporting qualitative research’ checklist used to guarantee uniformity in qualitative research based on interviews or focus groups [18].

### Population and setting

A purposive sample of 15 nursing students attending the postgraduate intensive care course at University of L’Aquila was enrolled during the academic year 2018/2019. Eligible students were contacted singularly face-to-face by the researchers to explain the aims of the study and obtain their consent to participate. Participant recruitment was performed until data saturation was reached [19].

### Data collection

Audio-recorded face-to-face in-depth interviews were held by a researcher in a dedicated room complemented with non-verbal communication outlined in the field notes. Interviews were performed through ad hoc open-ended questions (Table 2) and content was validated by researchers. The comprehensibility was tested on a small group of nurses not involved in the study. One week before performing the interviews, students were provided with the open-ended questions in order to allow time to both reflect on their experience and consequently, to bring out their most significant perceptions.

**Table 2:** Open-ended questions used for interviews

1.What is your opinion about the simulation experience based on multiple exposure to high-fidelity simulation?	
2.What thoughts/feelings did you experience before, during, and after this activity?	
3. How did this experience contribute to your learning and development of professional nursing competencies?	
4. What were your expectations about the high-fidelity simulation experience?	
5. Please share your thoughts on strength and weaknesses of using the high-fidelity simulation based on multiple exposure in training graduate students in critical care nursing	
6. Please provide suggestions on improving the high-fidelity simulation activity.	

### Analysis

Verbatim transcripts of recorded content were independently performed by two researchers to guarantee validity and reliability of data extraction. According to the phenomenological descriptive approach, the experience of participants was documented through the following steps: 1) bracketing, 2) intuiting, 3) analysing, and 4) describing [20]. In the first step, to avoid conditioning in the data analysis, researchers abstained from opinions, evaluations, and judgments about the phenomenon. In the second step, researchers read the text over and over to gain the deep sense of the lived experiences while considering the content of the field notes. Then, through a complete immersion in the content of interviews, researchers identified the essence of the phenomenon under investigation and the most significant statements were extracted. Finally, researchers captured the essential relationships among the statements to pull out themes representing the essence of the lived experiences. No software was used to handle the data.

### Ethical consideration

The study was carried out in accordance with the Declaration of Helsinki principles. Since HFS is regularly established within the curricula of the course, the Didactics and Ethics Board (DEB) of the Postgraduate Intensive Care Course gave its approval (04th March 2019). Before data collection, the study aims were explained to students, and they were asked to provide their voluntary written consent to data collection for which confidentiality in data management was guaranteed. Students voluntarily participated after being informed that their participation would not affect their academic pathway. No student has been forced to participate if there was a refusal.

## Results

Overall, 15 postgraduate students were enrolled. Nine (60.0%) were female and six (40.0%) were male and the mean age was 26.9 ± 3.9 years.

### Thematic analysis

The thematic analysis revealed a total of three main themes and ten categories (Table 3).

**Table 3:** Themes and categories from thematic analysis

Main themes	Categories
*Pragmatic learning experience*	1. Application of theory in practice
	2. Positive attitude
*Emotional path*	1. Anxiety
	2. Embarrassment
	3. Scepticism
	4. Comfort
	5. Safety
	6. Satisfaction
*Confidence*	1. Technical skills
	2. Non-technical skills

#### Pragmatic learning experience

Based on multiple exposures to HFS sessions, postgraduate students involved in this experience shared the perception of being immerged in an educational context that allowed them to put their theoretical knowledge into practice

“[ … ] *we enriched our knowledge by integrating theoretical concepts to the practice*” (interview #10)

“[ … ] *we put into practice all that we learned during lectures, obtaining knowledge and skills difficult to acquire in different modalities. At this regard, the practical component becomes fundamental*” (interview #4).

In addition, using multiple exposures to HFS sessions allowed them to gain a positive attitude towards this innovative didactic method

“[ … ] *the experience proved to be very effective for learning from both technical and non-technical points of view*” (interview #8)

not just because it was considered as useful but also, because it maximized learning through the repetition of a same experience

“[ … ] *The organization was excellent because gave us the opportunity to repeat simulation sessions for several times* [ … ] *after having detected our mistakes, we tried to deal with them by acting with further details and attention*” (interview #2).

#### The emotional path

With referral to the feelings experienced by students before, during, and after the simulation activities, following the repetitive cycle of this didactic experience, an emotional progression from negative to positive feelings was commonly described. Particularly, anxiety, embarrassment, and scepticism were the prevalent emotions reported at the beginning of the experience

“[ … ] *at first, I felt a little lost, anxious, and scared because I did not know what to expect from simulation*” (interview #10)

“[ … ] *the presence of a competent staff observing me during the sessions was an embarrassing component* [ … ]” (interview #5)

“[ … ] *at first, I was sceptic about the simulation experiences …* ” (interview #1).

In most cases, these emotions shifted to a feeling of comfort, likely due to the relationship with facilitators and the possibility of performing sessions repeatedly

*“*[ … ] *but the relationship with tutors and the progression of simulation sessions, turned my embarrassment into comfort*” (interview #5)

and to a sense of safety and satisfaction

“[ … ] *during the simulation activities, I felt trained because not all the illustrated procedures are usually performed in daily practice; some of them are rarely dealt with, therefore, having the possibility to both experience scenarios in a safe environment and have a tutor available allows to deal with the working reality with greater safety and awareness*” (interview #12)

“[ … ] *I was extremely satisfied from these experiences which were very educational and well-structured* [ … ]” (interview #8).

#### Confidence

The repetition of sessions appeared to improve students’ confidence in their technical skills

“[ … ] *during sessions, I felt my competences improved and, at the end I felt myself able perform almost all the technical skills* [ … ]” (interview #6).

and in their non-technical skills, such as teamwork abilities

“[ … ] *since simulations were performed in groups of three, it contributed to my professional training, mainly as regards teamwork. Therefore, I professionally grew in defining leadership and roles with the ultimate aim of rescuing the patient's life*” (interview #3).

and critical thinking

“[ … ] *I had the opportunity to review the appropriateness or not of my actions by means of both the directions given by the tutor and a better comprehension of theoretical rationale. These also contributed to the development of my critical thinking*” (interview #14).

### Students’ expectations, strengths, weaknesses, and future perspectives

Apart from collecting the lived experience of students towards the novel learning strategy in which they were involved, researchers also documented the expectations of students before entering the multiple simulation activities, their view about strengths and weaknesses of this method, and which future perspectives and expectations had to be met.

Some students reported low expectations towards multiple simulation activities that evolved into appreciation

“[ … ] *my expectations about simulations were not very high, I was also critic because I thought the laboratory was not provided with adequate equipment* [...] *I was very impressed with the laboratory and its realism*” (interview #4)

“*Initially my expectations were not positive* [...] *but by participating in the simulations and with the help of the tutor and support of colleagues, my expectations have become positive and I would have liked to repeat the session many other times* [ … ]” *(interview #3)*

others had high expectations and were not disappointed

“[ … ] *the expectations regarding simulations were high, thanks to the description made, and for what we have read about. Therefore, I thought it was an excellent opportunity for the improvement. Personally, the expectations have not been betrayed and I felt improved from this activity*” (interview #14).

Students considered the HFS a useful tool for their traineeship and career and agreed that the strengths of this learning method were the application of theory to practice and the possibility to self-evaluate their ability

“[ … ] *approaching directly with the clinical situation helped me to become confident, from the theoretical and practical point of view* [ … ]” (interview #13)

“[ … ] *one of the advantages of simulation consists in having higher awareness about our skills in the situations we could deal with* [ … ]” (interview #5)

“[ … ] *I think there are only advantages, and I think also this educational method could be spread to the rest of the nursing education* [ … ]” (interview #6)

“[ … ] *the advantages outweigh the disadvantages. The main advantages are that students are trained, and their skills are refined to give a better result also in the operational field* (interview #10).

About future perspectives of the novel learning method, students commonly agreed to increasing the amount of the simulation sessions

“[ … ] *I find it useful to increase the number of sessions* [ … ] (interview #2)

“[ … ] *increase the number of cases and the time dedicated to each activity* [ … ]” (interview #9).

## Discussion

During the 2018–2019 academic year, 15 students were enrolled in the study. Reflecting the national gender distribution of Italian nurses, most of them were female [21]. While, their mean age (26.9 years), was slightly higher than current graduates (24.6 years) in Italy [22].

To the researchers’ knowledge, this study is the first to report the lived experiences of postgraduate students participating to a novel learning experience based on multiple exposures to HFS. Knowing students’ reactions about the proposed teaching strategy helps faculty improve the quality of the learning process, review any phases considered problematic, and guarantee their efficacy. The thematic analysis highlighted that the learning experience played an important role in promoting a positive attitude in students about the novel teaching strategy. In this regard, multiple immersive experiences allowed students to apply the theoretical concepts in a safe and forgiving learning environment activating their perceptions, knowledge, and behaviours while the critical skills reinforcement, inspired by the principles of ‘learning for mastery’, improved their confidence [23–26]. The results concerning student’s appreciation for this learning activity are similar to those displayed in other educational settings in which simulation was considered an adequate preparation for caring for real patients in a suitable atmosphere [27–30]. This approach led to meeting students’ learning needs and times and allowed them to incorporate new operative models as well as technical and non-technical skills in the following HFS sessions [26, 31].

Multiple exposure to HFS, positive attitude, and confidence of students contributed to the transition from negative to positive feelings about the learning environment in which they had to simulate caring for a critically ill patient [25, 26, 32]. In fact, initially, students displayed a series of negative emotions such as anxiety, embarrassment, and scepticism toward the educational method, likely due to their clinical experience that could have affected how they perceived the realism of simulation sessions, or to the initial cognitive load related to an unfamiliar learning experience [25, 33]. Conversely, despite the initial doubts towards the proposed activities, all the students left the simulation experience with feelings of satisfaction, a sense of being enriched by the activity, and expressed appreciation towards the faculty tutors who made it possible to learn and practice with realistic clinical situations in which they were initially apprehensive to face in daily practice [34–36]. The novel experience was conducted in a safe and supportive learning environment in which students could feel free to make errors, talk about what they did not understand, repeat nursing techniques, and deepen their knowledge in the debriefing under the guidance of a facilitator. All these conditions could promote a sense of well-being in the students [34, 36, 37]. In this regard, the positive effect of fidelity and realism of scenarios on students’ learning experience has been confirmed [30]. Comfort and safety in the clinical management of critically ill patients were two other common feelings expressed by participants. They expressed their comfort in feeling trained, prepared and now ready to face critical clinical situations outside the simulated learning environment [38].

Reaching an adequate confidence level about technical and non-technical nursing skills is fundamental to promoting efficient nurse-to-patient relationships and good teamwork which are fundamental to guaranteeing patient safety in critical care areas [39]. In this regard, students commonly shared being more prepared to face critical situations in which they had to act, respond, and deliver several procedures to patients. Although groups involved in each HFS session were composed of students who had never worked together in stressful situations, the didactic approach based on the repetition of the learning experience seemed to overcome the difficulties of traditional simulation activities in which groups are briefly formed and then dissolved [40, 41]. Therefore, having taken part into the simulations as a team, students were made aware of the value of all members of the contributing clinical practice team [42]. The teamwork contribution for students is explored in another work, in which is documented that student took advantage from each other competencies, to solve the scenario, improving their teamwork abilities [43].

In accordance with the logic of the new educational model, students welcomed the increased number of simulation sessions. They thought this would improve their learning experience through the dilution of theoretical concepts over time, giving more opportunities for individual training, and assuring the effective achievement of all required nursing skills including critical thinking and clinical reasoning. The basic principle which allowed the achievement of these results was the scaffolding process by which faculty created learning opportunities with increasing complexity, providing an educational framework for learning by which students attempted to reach new knowledge and skills through their own initiative, motivation, and resourcefulness [44, 45]. In this framework, training based on multiple exposures to HFS focalized on specific educational needs of postgraduate students may represent an effective method to guarantee the acquisition of advanced technical and non-technical nursing skills.

Finally, according to previous research experiences that involved postgraduate nursing students, participants were highly satisfied by the simulation experience [14, 46].

### Limits, strength, and future perspectives

In this study, was adopted a single-site phenomenological descriptive design which does not allow for any generalization of results in other contexts. However, the lived experiences of intensive care nursing students exposed to a new model of HFS training was investigated for the first time. This may be a valuable contribution to the international debate related to the effectiveness of HFS training in promoting postgraduate nursing students learning outcomes.

According to Kirkpatrick model, knowing students’ reactions is a first step to improve the quality of training through a deep revision of the learning processes. However, the effectiveness of the proposed model must be further investigated in future studies.

## Conclusion

The novel approach applied in this study has been lived as a pragmatic learning experience by postgraduate students enhancing their ability to apply theory to practice and develop a positive attitude towards HFS. Multiple exposure to HFS contributed to the transition from negative to positive feelings about the learning environment in which they had to simulate caring for a critically ill patient. At the end of all the simulation experiences students perceived an improvement in their confidence about technical and non-technical skills when caring for a critically ill patient.

## Data Availability

The dataset generated and/or analysed during the current study are not publicly available due to promises of participant anonymity and confidentiality but are available from the corresponding author on reasonable request.

## Abbreviation

HFS: High-fidelity simulation
